# Efficacy of a technology-based client-centred training system in neurological rehabilitation: a randomised controlled trial

**DOI:** 10.1186/s12984-021-00977-2

**Published:** 2021-12-28

**Authors:** Els Knippenberg, Annick Timmermans, Jolijn Coolen, Katrien Neven, Peter Hallet, Jolien Lemmens, Annemie Spooren

**Affiliations:** 1 Department of Healthcare, Centre of Expertise – Innovation in Care, PXL University of Applied Sciences and Arts, Guffenslaan 39, 3500 Hasselt, Belgium; 2 Faculty of Rehabilitation Sciences, REVAL, Hasselt University, Agoralaan, Gebouw A, 3590 Diepenbeek, Belgium; 3 Noorderhart, Rehabilitation and MS Center, Boemerangstraat 2, 3900 Pelt, Belgium; 4 St-Trudo Hospital, Diestersteenweg 100, 3800 St-Truiden, Belgium; 5 Ziekenhuis Oost-Limburg, Campus Sint-Barbara, Bessemerstraat 478, 3620 Lanaken, Belgium; 6 Sint-Gerardus, Multifunctioneel Centrum, Sint-Gerardusdreef 1, 3590 Diepenbeek, Belgium

**Keywords:** Client-centred, Task-oriented, Rehabilitation, Technology, Efficacy

## Abstract

**Background:**

A client-centred task-oriented approach has advantages towards motivation and adherence to therapy in neurorehabilitation, but it is costly to integrate in practice. An intelligent Activity-based Client-centred Training (i-ACT), a low-cost Kinect-based system, was developed which integrates a client-centred and task-oriented approach. The objectives were (1) to investigate the effect of additional i-ACT training on functioning. And (2) to assess whether training with i-ACT resulted in more goal oriented training.

**Methods:**

A single-blind randomised controlled trial was performed in 4 Belgian rehabilitation centres with persons with central nervous system deficits. Participants were randomly allocated through an independent website-based code generator using blocked randomisation (n = 4) to an intervention or control group. The intervention group received conventional care and additional training with i-ACT for 3 × 45 min/week during 6 weeks. The control group received solely conventional care. Functional ability and performance, quality of life (QoL), fatigue, trunk movement, and shoulder active range of motion (AROM) were assessed at baseline, after 3 weeks and 6 weeks of training, and 6 weeks after cessation of training. Data were analysed using non-parametric within and between group analysis.

**Results:**

47 persons were randomised and 45 analysed. Both intervention (n = 25) and control (n = 22) group improved over time on functional ability and performance as measured by the Wolf Motor Function Test, Manual Ability Measure-36, and Canadian Occupational Performance Measure, but no major differences were found between the groups on these primary outcome measures. Regarding QoL, fatigue, trunk movement, and shoulder AROM, no significant between group differences were found. High adherence for i-ACT training was found (i.e. 97.92%) and no adverse events, linked to i-ACT, were reported. In the intervention group the amount of trained personal goals (88%) was much higher than in the control group (46%).

**Conclusions:**

Although additional use of i-ACT did not have a statistically significant added value regarding functional outcome over conventional therapy, additional i-ACT training provides more individualised client-centred therapy, and adherence towards i-ACT training is high. A higher intensity of i-ACT training may increase therapy effects, and should be investigated in future research.

*Trial registration:* ClinicalTrials.gov Identifier NCT02982811. Registered 29 November 2016.

## Background

Rehabilitation in persons with central nervous system (CNS) deficits such as stroke, multiple sclerosis, and spinal cord injury, is important to regain and/or maintain functional ability in activities of daily life (ADLs), and consequently optimise quality of life (QoL) [1–5]. Practice methods that showed promising results regarding motivation and effectiveness in neurorehabilitation, are task-oriented therapy and client-centred training [6–11]. Task-oriented training is considered a highly individualised training of functional tasks based on task segmentation [6, 7]. The client-centred approach incorporates the person’s wishes and needs, and actively involves the person with deficits in setting certain goals in their rehabilitation process [7, 9–12]. By using occupational models and assessments, such as the Person-Environment-Occupation model (PEO-model) [13] and Canadian Occupational Performance Measure (COPM) [14–16], therapists can involve the person with deficits in the process of setting unique and individual goals, which increases therapy motivation and consequently adherence. The extra advantage of the COPM is that it cannot only be used for goal setting but also for the assessment of self-perceived occupational performance [14–16].

Although motivation is higher when using a task-oriented client-centred approach, in practice this is time-consuming. To increase persons’ motivation and treatment adherence, technology-based systems with immersive or non-immersive virtual reality (VR) or augmented reality (AR) such as Nintendo Wii or Microsoft Kinect, can be used [1, 2, 5, 17–20]. However, these commercially available, low-cost systems do not incorporate a client-centred task-oriented approach, and the standard (exer)games are not developed to meet the rehabilitation goals such as improving coordination patterns or compensation strategies when performing task-oriented exercises [1, 2, 4, 5, 21]. Although these systems are not developed to meet rehabilitation goals, we explored the skeleton tracking feature of Microsoft Kinect in earlier research and developed an intelligent activity-based client-centred training (i-ACT) system using Microsoft Kinect sensor and software development kit [22]. i-ACT allows persons with CNS deficits to train more explicit on individual goals and the usability of i-ACT and persons’ motivation, credibility and expectancy towards using i-ACT for rehabilitation purposes, was established [23]. Results of that cohort study showed an increase over time regarding upper limb functional ability and perceived performance, but no comparison was made with a control group [24]. The purpose of this trial was to investigate the effect of additional i-ACT training on functional ability, occupational performance, quality of life (QoL), fatigue, trunk movement, and shoulder active range of motion (AROM) compared to conventional therapy alone. Our first hypothesis was that there is a positive effect of additional i-ACT training on functional ability and perceived occupational performance in comparison with conventional therapy. Our secondary hypothesis was that as compared to conventional therapy, there is a positive effect of additional i-ACT training on quality of life, fatigue, trunk impairment and AROM in the shoulder. The third hypothesis was that the individualised goals set by persons with deficits are trained more explicit when exercising with i-ACT compared to conventional therapy.

## Methods

### Participants and study protocol

Persons with CNS were recruited in four rehabilitation centres in Flanders (Belgium) to participate in a single-blind randomised controlled trial (RCT).

The inclusion criteria were: age over 18 years old, a medical diagnosis of central nervous system disease, and dysfunction in upper limb and/or core stability. Persons with multiple sclerosis (MS) had to be free of treatment with corticosteroids for one month. Persons with stroke or spinal cord injury, had to be at least three months post injury. Exclusion criteria were: severe spasticity (when spasticity impedes movement), severe cognitive impairment (person is not able to understand and follow instructions), severe communicative impairment (person is not able to answer questions), severe visual impairment (person is not able to see the television screen), persons who use an electric wheelchair as the Microsoft Kinect® might have troubles recognising a human skeleton.

Potential participants were recruited by either the rehabilitation physician or therapist based on the person with deficits’ medical files. Information letters about the study and an invitation to participate were provided to the potential participants. Furthermore, an individual meeting was scheduled with these potential participants to provide answers to possible questions or concerns. During this individual meeting, the potential participants were screened by the primary supervisor as to the inclusion and exclusion criteria. After receiving informed consent, the COPM was conducted to collect the participants’ individual goals towards rehabilitation. These goals were discussed with the occupational and/or physiotherapist of the individual participant as to what extend these goals were realistic and relevant, and generally in accordance with the therapeutic goals.

After inclusion, the participants were randomly allocated to either the experimental group (i-ACT training with conventional care) or the control group (conventional care), using blocked randomisation (block size n = 4). The randomisation procedure was performed by an independent researcher (JL) using the website www.sealedenvelope.com/simple-randomiser from which also a unique code per participant was generated. The primary researcher (EK) involved in data collection was blinded for the group allocation.

Outcomes measures were collected by the primary researcher at baseline (T_0_), after 3 weeks (T_1_) and 6 weeks (T_2_) of training, and at 6 weeks follow-up (T_3_). To provide a stable image of the participant, baseline measures were performed 3 times over 3 weeks.

Participants received 3 × 45 min of training with i-ACT (see Apparatus) during six weeks, additional to conventional care. Exercises in i-ACT were individually set to meet the individual goals of the participants as set by the COPM and discussed with the person’s therapists. Furthermore, exercises were individually set regarding possibilities and progression of each individual participant.

### Outcome measures

The following *demographic data* were obtained from the participant or medical files: age, gender, diagnosis, and time of diagnosis.

The *primary outcome measures* were the Wolf Motor Function Test (WMFT) [25–27], Manual Ability Measure-36 (MAM-36) [25, 26, 28, 29], and Canadian Occupational Performance Measure and COPM [14, 15]. The secondary outcome measures were Short Form-36 (SF-36) [30], Modified Fatigue Impact Scale (MFIS)[31–33], Trunk Impairment Scale (TIS)[34–36], and Active Range of Motion (AROM) of the shoulder.

The WMFT is a test for arm-hand functioning on International Classification of Functioning, Disability and Health (ICF) level of actual performance (activity level). The WMFT contains 17 items (2 strength-based tasks and 15 function-based tasks) arranged in order of complexity. The strength-based tasks are measured by weight lift and grip strength, while the 15 function-based tasks are timed and scored on a scale from 0 (not able to perform task) to 5 (normal performance) [26, 27, 37]. The MAM-36 is a questionnaire on International Classification of Functioning, Disability and Health (ICF) level of perceived occupational performance (activity level). Questions relate to the ease or difficulty level of how a person is able to perform unilateral and bilateral ADL-tasks. Scores range from 0 (impossible) to 4 (easy) [25, 26, 28, 29]. The COPM is a client-centred individualised instrument on ICF Participation level. This outcome measure is developed to capture a person’s self-perception of performance in ADL, over time. By means of a semi-structured interview, persons are asked to identify their 5 main goals in self-care, productivity and/or leisure. These 5 goals are scored on execution and satisfaction with scores ranging from 0 (negative) to 10 (positive) [15, 16, 38, 39].

*Secondary outcome measures* were the SF-36 [30], MFIS [31–33], TIS [34–36], and AROM of the shoulder.

The SF-36 is a 36-item, person-reported survey regarding QoL measures, on all ICF levels (i.e. ICF Function, ICF Activity, and ICF Participation)[40]. The SF-36 consists of 8 categories, i.e. physical functioning, role limitations due to physical health, role limitations due to emotional problems, energy/fatigue, emotional well-being, social functioning, pain, and general health. The higher the score, the more favourable health state is reported [30, 41–44]. The MFIS is a questionnaire which provides information on how fatigue impacts the life of the person, in terms of physical, cognitive, and psychosocial functioning. Twenty-one items are scored on a 5-point Likert-scale (range from 0 = never to 4 = almost always) [31–33]. The MFIS is not specified to one domain of the ICF. The TIS is an assessment to measure motor impairment of the trunk by evaluating 3 aspects: static sitting balance, dynamic sitting balance, and trunk co-ordination. Each item is scored on a 2-, 3- or 4-point scale, ranging from a minimum of 0 to a maximum of 23 points [34–36]. The TIS is an assessment in ICF level of actual performance (activity level). The AROM refers to the possible range in motion while performing non-assisted voluntary movement of a body part on ICF function level. In this study, the AROM of the shoulder joint is measured with a goniometer for abduction and flexion in relation to the torso from the neutral anatomical position.

### Ethics statement

All study procedures were approved by the Medical Ethics Committee of UZ KU Leuven (Registration number B322201731417) and local Ethics Committees of the participating centres. All participants signed an informed consent prior to participating in the study. The clinical trial was registered as NCT02982811.

### Apparatus

i-ACT consists of the Microsoft Kinect® sensor and the Microsoft Kinect® software development kit (SDK). The Microsoft Kinect® detects a human shape and human movements. For the technical development of i-ACT, the cross-platform Unity3D was used. The most important feature of i-ACT is that the client-centred approach is involved in every step, as well as the possibility to work task-oriented. Therapists can record a movement which is valuable for the person with deficits, then can set up the necessary parameters to progress towards an exercise (e.g. amount of repetitions, target placement, etc.) which is unique for this specific participant. Furthermore, one or more stability points can be integrated. A point of stability is a body region (joint) that is marked with a coloured sphere as an area where no movement is allowed beyond a certain bandwidth, i.e. to avoid compensatory movement. The therapists can set the stability point to one or more joints and can also adapt the size of the sphere. The bigger the sphere, the more (compensational) movement is allowed in this joint. When the person with deficits moves out of the sphere, the person receives feedback that the movement is not correct.

**Fig. 1:**
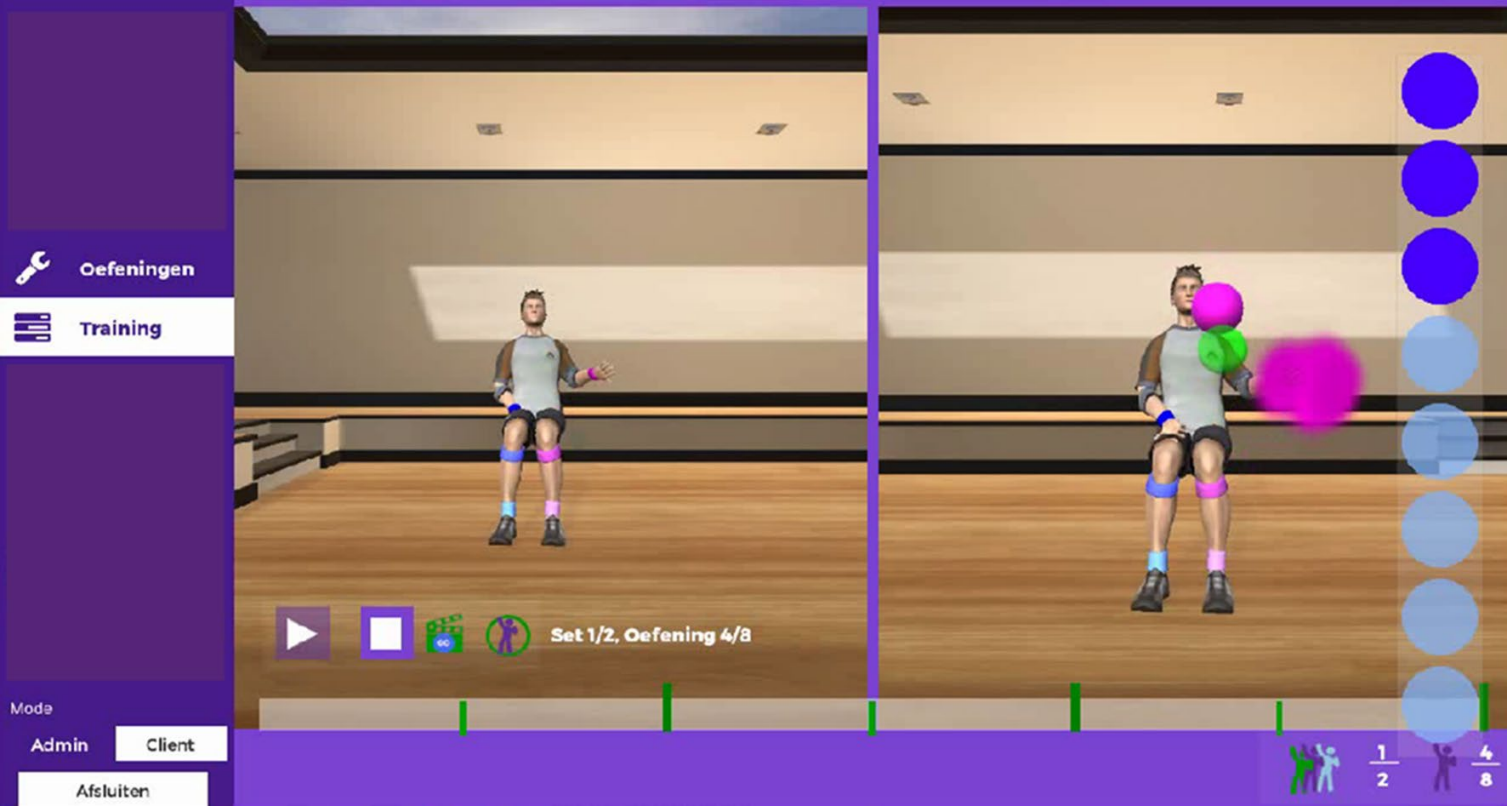
Person with deficits’ interface during performance of the exercise “drinking from a cup”. The avatar on the left represents the therapist (i.e. recorded movement) while the avatar on the right represents the person with deficits. The green dot is the stability point for restriction of compensational movement. The pink dots are the targets for the right hand

The person with deficits is visualised by an avatar in a virtual environment where the person receives real time feedback on successful trajectories and stabilisation of set body areas, i.e. quality of movement (see Fig. 1). A more detailed description of the development of i-ACT is explained in Knippenberg et al. [22].

### Statistical analysis

If data did not follow a normal distribution pattern, non-parametric statistics were used.

For differences within groups, a Friedman’s ANOVA was performed to assess if significant progress was made over time (baseline T0, after 3 weeks of training T1, after cessation of training T2, and after 6 weeks follow-up T3). The Wilcoxon signed-rank test was performed to assess significant progress over time between baseline and cessation of training (i.e. training period T0-T2), and cessation of training and follow-up (follow-up period T2-T3). Alpha was set at 0.05 and a Bonferroni approach was used. The Bonferroni corrected alpha value equals 0.025 for data comparison between T0-T2 and T2-T3. When significant difference was found in the Wilcoxon signed-rank test, the effect size *r* was calculated to look at the levels of change according to the Cohen’s benchmarks (i.e. *r* between 0.3 and 0.5 for a medium effect, and *r* above 0.5 for a large effect) [45]. Differences between i-ACT intervention group and control group were tested with the Mann–Whitney *U* test. The data of the persons who dropped out or were lost to follow-up, were treated as missing data and as such analysed using pairwise deletion. By using pairwise deletion, we preserved more information in relation to listwise deletion. Data were analysed using SPSS software (SPSS Inc., Chicago, IL). Differences between intervention group and control group regarding distribution of trained versus untrained goals was performed using descriptive analysis using Microsoft Excel (2016).

## Results

**Fig. 2:**
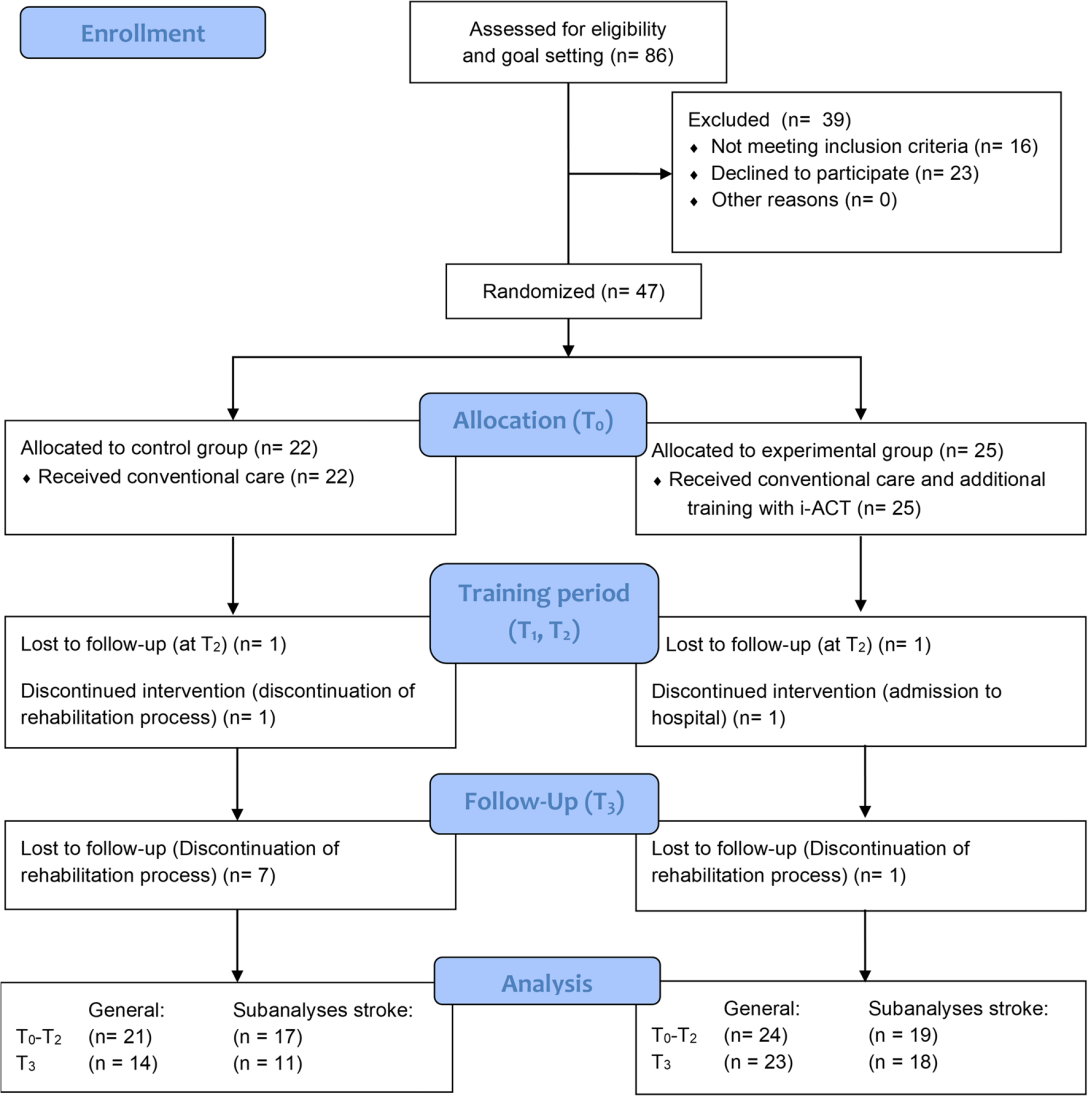
Flow chart (CONSORT). *T0* Baseline, *T1* after 3 weeks of training, *T2* test moment after 6 weeks of training, *T3* test moment 6 weeks after cessation of training

Figure 2 represents a flow chart of the study. A total of 47 persons were allocated using a block randomisation per centre per 4 participants. Forty-five persons completed the training period and 37 persons completed the full protocol. As 80% of the participants were diagnosed with stroke (36 out of 45), the same statistical post hoc analyses were performed for persons with stroke, to look into possible differences between the general group (i.e. all diagnoses included, n = 45) and stroke group (i.e. persons with stroke, n = 36). Two persons dropped out during the training period due to discharge from the hospital. The inclusion of participants started in March 2017 and lasted until May 2020.

### Participants characteristics

Participants characteristics are shown in Table 1. In total, 45 participants were included for the analyses of which 27 male and 18 female persons with CNS disease, with a mean age of 59.07 ± 16.42. Twenty-one participants of which 11 males and 10 females, were allocated in the control group, mean age 60.14 ± 16.72. In the intervention group, 24 participants were allocated of which 16 males and 8 females, with a mean age of 58.13 ± 16.46. The difference in number of the allocated participants between control (n = 21) and intervention group (n = 24) is due to the use of block randomisation (n = 4) by participating rehabilitation centre (n = 4). All baseline characteristics and outcome measures deviated from normal distribution for both control group and intervention group, except for age (p = 0.053 and 0.656 respectively). Regarding primary outcome measures at baseline, only a significant difference between intervention and control group was found in MAM-36 (p = 0.036) and COPM-satisfaction (p = 0.036). Compliance of participants with attending the intervention sessions was 97.92%. Two participants missed 9 sessions of which 6 sessions were missed due to doctor appointments / hospital visits, 3 sessions were missed due to external family-related activities (i.e. family birthday party). No adverse effects of the intervention were found.

**Table 1:** Participants characteristics

	Intervention group (n = 24)	Control group (n = 21)	Between group difference
Gender (m/v)	16/8	11/10	
Age	58.13 ± 16.46	60.14 ± 16.72	p = 0.569
Diagnosis (n)			p = 0.757
Stroke	19	17	
MS	2	2	
Other	3^a^	2^b^	
Time since diagnosis (months)	13.25 ± 22.83	16.00 ± 41.32	p = 0.376
Time (hours/week)			
Conventional therapy	8.33 ± 3.51	7.62 ± 3.58	p = 0.431
Intervention exercise	1.47 ± 0.02	NA	
WMFT			
FAS	47.96 ± 10.73	48.67 ± 12.01	p = 0.864
Time (seconds)	120.01 ± 131.53	102.00 ± 132.04	p = 0.246
MAM-36	88.29 ± 32.66	107.90 ± 18.61	p = 0.036* (r = − 0.312)
COPM			
Performance	22.38 ± 9.53	17.52 ± 8.22	p = 0.077
Satisfaction	23.79 ± 11.706	16.90 ± 9.47	p = 0.036* (r = 0.312)

Data presented as mean ± SD unless mentioned otherwise; *Significant difference with Mann–Whitney *U*-test *p*-value and effect size r; *MS* Multiple Sclerosis, *WMFT* Wolf Motor Function Test, *FAS* Functional Ability Scale, *MAM-36* Manual Ability Measure-36, *COPM* Canadian Occupational Performance Measure, *NA* not applicable

Other diagnosis: ^a^Guillain-Barré (n = 1), Spinal Cord Injury (n = 1), and Parkinson's disease (n = 1); ^b^Amyotrophic Lateral Sclerosis (n = 1), and brain surgery (n = 1)

### Outcome measures

An overview of the test results can be found in Table 2. The mean delta scores (i.e. cessation of training values minus baseline values and follow-up values minus cessation of training values) are represented in Table 3.

**Table 2:** Results on outcome measures

	Intervention group				Within group difference		Control group				Within group differences	
	T0	T1	T2	T3	α ≤ 0.025		T0	T1	T2	T3	α ≤ 0.025	
	n = 24	n = 24	n = 24	n = 23	T0-T2	T2-T3	n = 21	n = 21	n = 21	n = 14	T0-T2	T2-T3
WMFT												
Functional ability	52.00 (39.50–55.00)	51.50 (45.25–59.75)	55.50 (49.25–63.00)	59.00 (51.00–67.00)	**p = 0.000**	**p = 0.000**	49.00 (43.50–55.50)	51.00 (44.00–62.00)	55.00 (45.00–63.50)	57.50 (51.00–68.75)	**p = 0.000**	p = 0.066
Time	64.28 (47.65–135.99)	54.11 (41.84–88.91)	53.60 (37.23–85.79)	42.03 (36.01–69.13)	**p = 0.003**	**p = 0.002**	52.20 (36.12–102.54)	45.63 (29.03–77.19)	43.00 (28.22–82.82)	42.95 (30.90–63.14)	**p = 0.004**	p = 0.050
MAM-36	93.50 (68.50–112.75)	107.00 (79.50–124.50)	112.00 (95.25–130.00)	110.00 (81.00–132.00)	**p = 0.000**	p = 0.711	105.00 (94.50–125.50)	108.00 (97.00–129.50)	107.00 (93.50–128.50)	122.00 (103.00–135.75)	p = 0.204	**p = 0.016**
COPM												
Performance	24.00 (14.25–29.75)	NA	29.00 (22.75–33.00)	30.00 (21.00–36.00)	**p = 0.001**	p = 0.041	18.00 (10.50–23.00)	NA	20.00 (16.50–30.00)	25.00 (18.50–34.50)	**p = 0.008**	**p = 0.011**
Satisfaction	24.50 (15.75–31.00)	NA	29.50 (21.25–35.75)	30.50 (19.50–39.50)	**p = 0.002**	p = 0.059	15.00 (8.00–24.00)	NA	20.00 (16.00–27.50)	29.00 (19.00–39.00)	**p = 0.005**	p = 0.085
SF-36												
Health change	25.00 (0.00–43.75)	25.00 (0.00–50.00)	25.00 (6.25–50.00)	25.00 (0.00–75.00)	p = 0.178	p = 0.596	25.00 (0.00–50.00)	25.00 (0.00–50.00)	50.00 (0.00–50.00)	25.00 (0.00–56.25)	p = 0.305	p = 0.655
Physical functioning	40.00 (21.25–66.25)	47.50 (31.25–81.25)	52.50 (36.25–81.25)	45.00 (30.00–80.00)	p = 0.056	p = 0.913	55.00 (30.00–70.00)	50.00 (30.00–75.00)	55.00 (30.00–80.00)	65.00 (30.00–81.25)	p = 0.586	p = 0.161
Role functioning/physical	25.00 (0.00–50.00)	37.50 (0.00–68.75)	25.00 (0.00–68.75)	25.00 (0.00–75.00)	p = 0.977	p = 0.415	25.00 (0.00–62.50)	25.00 (12.50–50.00)	25.00 (0.00–50.00)	50.00 (0.00–75.00)	p = 0.273	p = 0.157
Role functioning/emotional	100.00 (67.00–100.00)	100.00 (66.70–100.00)	100.00 (33.33–100.00)	100.00 (66.70–100.00)	p = 0.504	p = 0.452	100.00 (50.00–100.00)	100.00 (33.30–100.00)	100.00 (33.30–100.00)	100.00 (33.30–100.00)	p = 0.397	p = 0.527
Energy/fatigue	67.50 (60.00–80.00)	70.00 (50.00–80.00)	62.50 (41.25–83.75)	70.00 (50.00–85.00)	p = 0.269	p = 0.046	60.00 (47.50–75.00)	65.00 (47.50–75.00)	65.00 (47.50–80.00)	70.00 (53.75–85.00)	p = 0.435	p = 0.226
Emotional well-being	78.00 (68.00–91.00)	80.00 (53.00–92.00)	76.00 (49.00–88.00)	80.00 (56.00–92.00)	p = 0.137	p = 0.057	72.00 (64.00–86.00)	80.00 (60.00–86.00-	80.00 (62.00–90.00)	88.00 (63.00–100.00)	p = 0.302	p = 0.306
Social functioning	75.00 (63.00–97.00)	87.50 (62.50–100.00)	87.50 (62.50–100.00)	87.50 (62.50–100.00)	p = 0.146	p = 0.836	75.00 (44.00–88.00)	62.50 (62.50–87.50)	62.50 (50.00–87.50)	68.75 (50.00–78.13)	p = 0.574	p = 0.932
Pain	68.00 (47.50–79.50)	73.75 (47.50–89.38)	70.00 (55.63–90.00)	67.50 (55.00–90.00)	p = 0.284	p = 0.649	68.00 (46.50–95.00)	67.50 (45.00–100.00)	77.50 (45.00–100.00)	77.50 (67.50–100.00)	p = 0.875	p = 0.733
General health	55.00 (45.00–73.75)	62.50 (35.00–88.75)	57.50 (45.00–83.75)	50.00 (45.00–75.00)	p = 0.214	p = 0.188	45.00 (30.00–72.50)	60.00 (37.50–75.00)	55.00 (35.00–72.50)	50.00 (27.50–73.75)	p = 0.951	p = 0.305
MFIS	27.00 (10.50–36.00)	22.00 (10.00–40.00)	25.50 (7.75–39.75)	24.00 (6.00–35.00)	p = 0.891	p = 0.570	28.00 (20.50–37.50)	31.00 (19.00–36.50)	33.00 (17.00–39.00)	20.50 (10.75–36.50)	p = 0.809	p = 0.514
TIS	15.00 (13.00–19.00)	17.50 (16.00–21.75)	20.50 (17.00–22.00)	22.00 (16.00–23.00)	**p = 0.000**	p = 0.306	14.00 (11.50–18.50)	17.00 (14.00–20.50)	17.00 (13.50–23.00)	21.00 (16.75–23.00)	**p = 0.002**	p = 0.102
AROM												
Abduction	86.50 (66.25–124.75)	89.00 (75.50–137.00)	97.50 (82.25–131.00)	112.00 (85.00–148.00)	**p = 0.014**	**p = 0.025**	118.00 (73.50–146.00)	119.00 (68.00–145.00)	114.00 (70.00–140.50)	132.00 (103.50–152.50)	p = 0.296	p = 0.298
Flexion	98.50 (78.25–126.75)	107.50 (87.75–130.50)	108.50 (91.75–131.75)	118.00 (100.00–137.00)	**p = 0.011**	p = 0.046	120.00 (88.50–145.00)	122.00 (107.50–143.00)	123.00 (101.00–141.50)	130.00 (120.75–159.00)	p = 0.149	**p = 0.006**

Data represented as median (interquartile range). *p*-values from Wilcoxon signed-rank test (2-sided-, significant p-values are indicated in bold font. *WMFT* Wolf Motor Function Test, *FAS* Functional Ability Scale, *MAM-36* Manual Ability Scale, *COPM* Canadian Occupational Performance Measure, *SF-36* Short Form-36, *MFIS* Modified Fatigue Impact Scale, *TIS* Trunk Impairment Scale, *AROM* Active Range of Motion

**Table 3:** Median (interquartile range) delta scores during training period and follow-up period with between group difference

	Intervention group		Control group		Between group difference	
	ΔT2-T0 MED (IQR)	ΔT3-T2 MED (IQR)	ΔT2-T0 MED (IQR)	ΔT3-T2 MED (IQR)	ΔT2-T0	ΔT3-T2
WMFT						
FAS	6.67 (1.67–11.83)	3.00 (0.00–6.00)	4.00 (1.00–7.50)	0.50 (-0.25–6.75)	p = 0.168	p = 0.270
Time	− 10.37 (− 33.89–− 4.10)	− 4.00 (− 15.00–0.00)	− 8.01 (− 31.62–− 3.15)	− 6.00 (− 16.00–0.00)	p = 0.909	p = 0.938
MAM-36	14.83 (7.17–27.17)	0.00 (− 5.00–6.00)	3.67 (− 2.17–9.75)	4.50 (0.00–8.75)	p = 0.000*	**p = 0.031**^**£**^
COPM						
Performance	6.50 (2.00–11.75)	2.00 (0.00–4.00)	4.00 (− 0.50–9.00)	2.50 (1.00–6.25)	p = 0.161	p = 0.298
Satisfaction	6.00 (1.50–9.75)	1.00 (0.00–4.00)	4.00 (1.00–8.50)	1.00 (− 0.25–4.25)	p = 0.465	p = 0.938
SF-36						
Health change	0.00 (0.00–25.00)	0.00 (0.00–0.00)	0.00 (0.00–25.00)	0.00 (0.00–0.00)	p = 0.672	p = 0.938
Physical functioning	5.00 (− 3.75–22.50)	0.00 (− 15.00–10.00)	0.00 (− 12.50–17.50)	0.00 (− 5.00–0.00)	p = 0.378	p = 0.344
Role functioning/physical	0.00 (− 25.00–25.00)	0.00 (− 25.00–25.00)	0.00 (− 25.00–0.00)	0.00 (0.00–25.00)	p = 0.486	p = 0.699
Role functioning/emotional	0.00 (0.00–0.00)	0.00 (0.00–0.00)	0.00 (− 16.67–0.00)	0.00 (− 8.25–8.25)	p = 0.728	p = 0.817
Energy/fatigue	− 5.00 (− 10.00–8.75)	0.00 (0.00–25.00)	0.00 (− 7.50–12.50)	0.00 (− 7.50–0.00)	p = 0.180	p = 0.042*
Emotional well-being	0.00 (− 15.00–7.00)	4.00 (0.00–8.00)	4.00 (− 2.00–8.00)	0.00 (0.00–5.00)	p = 0.139	p = 0.526
Social functioning	0.00 (0.00–12.50)	0.00 (0.00–0.00)	0.00 (− 12.50–12.50)	0.00 (− 13.00–3.25)	p = 0.347	p = 0.793
Pain	10.00 (− 9.38–18.13)	0.00 (− 10.00–20.00)	0.00 (− 7.50–11.25)	0.00 (0.00–13.00)	p = 0.490	p = 0.963
General health	5.00 (− 5.00–13.75)	0.00 (− 5.00–0.00)	− 5.00 (− 10.00–10.00)	0.00 (− 6.25–0.00)	p = 0.314	p = 0.914
MFIS	− 0.67 (− 6.50–6.00)	0.00 (− 10.00–3.00)	0.00 (− 5.50–7.00)	0.00 (− 5.75–1.25)	p = 0.759	p = 0.865
TIS	3.67 (2.17–5.83)	0.00 (0.00–2.00)	3.00 (0.50–4.67)	0.00 (0.00–0.50)	p = 0.274	p = 0.817
AROM						
Abduction	14.33 (− 4.96–19.50)	6.00 (− 3.00–18.00)	6.50 (− 2.67–13.17)	1.00 (− 2.50–6.25)	p = 0.127	p = 0.147
Flexion	8.17 (0.75–24.92)	4.00 (0.00–17.00)	5.50 (− 3.50–13.50)	5.50 (0.75–11.25)	p = 0.387	p = 0.963

Data represented as median (interquartile range). *Δ* Delta score, *MED* median, *IQR* Interquartile range, significant p-values regarding between group differences are indicated in bold font, *WMFT* Wolf Motor Function Test, *FAS* Functional Ability Scale, *MAM-36* Manual Ability Scale, *COPM* Canadian Occupational Performance Measure, *SF-36* Short Form-36, *MFIS* Modified Fatigue Impact Scale, *TIS* Trunk Impairment Scale, *AROM* Active Range of Motion; *Significant between group difference in favour of intervention group; ^£^Significant between group difference in favour of control group

### Within group differences

Significant differences (p < 0.05) within intervention and control group were found over the total time in all the primary outcome measures, i.e. WMFT, MAM-36 and COPM. Furthermore, significant differences (p < 0.05) within both groups over time were also found in TIS and AROM-flexion. Within the intervention group, a significant difference (p < 0.05) over time was found in AROM-abduction, while in the control group, a significant difference (p < 0.05) over time was found in SF-36 subscale emotional well-being. No significant differences were found in either group for MFIS.

When examining the specific period of training period (T0-T2) and follow-up (T2-T3), the significant differences are, as expected, mainly found in the training period, as seen in Table 2. Regarding the training period (T0-T2), significant improvement was found in the intervention group in all primary outcome measures, i.e. WMFT-FAS (p = 0.000), WMFT-Time (p = 0.003), MAM-36 (p = 0.000), COPM-performance (p = 0.001), and COPM-satisfaction (p = 0.002), and secondary outcome measures TIS (p = 0.000), AROM-abduction (p = 0.014), and AROM-flexion (p = 0.011). In the control group, significant improvement was found in 2 out of 3 primary outcome measures, i.e. WMFT-FAS (p = 0.000), WMFT-Time (p = 0.004), COPM-performance (p = 0.008), and COPM-satisfaction (p = 0.005), and in 1 secondary outcome measure (TIS with p = 0.002).

Examining the follow-up period (T2-T3), significant improvement is found in the intervention group for WMFT-FAS (p = 0.000), WMFT-Time (p = 0.002), and AROM-abduction (p = 0.025). In the control group, significant improvement is found during the same period in MAM-36 (p = 0.016), COPM-performance (p = 0.011), and AROM-flexion (p = 0.006).

No significant differences in training period as well as follow-up period were found in SF-36 and MFIS in both intervention or control group.

### Between group differences

An overview of the delta scores between baseline and cessation of training (T2), and cessation of training (T2) and follow-up (T3) can be found in Table 3. With regard to the primary outcomes, only a significant improvement was found for MAM-36 in training period in favour of intervention group (p = 0.000 with r = 0.561). For the follow-up period, this significant improvement was in favour of the control group (p = 0.031 with r = -0.358). Regarding the secondary outcome measures, only a significant improvement in favour of the intervention group was found in SF-36 energy/fatigue subscale for the follow-up period (p = 0.042, r = 0.340).

### Individual goal setting

Although the expectation was that a significant difference would occur between intervention and control group with regards to COPM, no significant difference was found. Therefore, a distribution of untrained versus trained goals was performed. An overview of the distribution of untrained versus trained goals in both control and intervention group can be found in Table 4. In general, in the control group, the distribution of untrained versus trained is about the same (i.e. 50%). While in the intervention group, it is clear that more than 85% of the participants’ chosen goals, were implemented in the therapy as usual together with the additional therapy with i-ACT.

**Table 4:** Percentages of untrained versus trained COPM goals

COPM goals	Control group (%)	Intervention group (%)
Untrained	54.29	12.50
Trained	45.71	87.50

*COPM* Canadian Occupational Performance Measure

### Post hoc* analyses*

As the majority of participants were persons with stroke, a post hoc analyses was performed for persons with stroke. An overview of the test results is presented in Table 5.

**Table 5:** Results on outcome measures from post hoc analyses in persons with stroke

	Intervention group				Within group difference		Control group				Within group difference	
	T0	T1	T2	T3	α ≤ 0.025		T0	T1	T2	T3	α ≤ 0.025	
	n = 19	n = 19	n = 19	n = 18	T0-T2	T2-T3	n = 17	n = 17	n = 17	n = 11	T0-T2	T2-T3
WMFT												
Functional ability	53.00 (38.00–55.00)	52.00 (45.00–60.00)	54.00 (50.00–63.00)	58.00 (52.50–66.00)	**p = 0.000**	**p = 0.002**	49.00 (41.00–55.50)	53.00 (44.00–62.50)	51.00 (43.50–64.00)	62.00 (51.00–68.00)	**p = 0.002**	p = 0.127
Time	74.83 (47.93–137.13)	58.82 (43.60–97.15)	54.61 (37.49–87.85)	41.24 (36.60–71.63)	**p = 0.010**	**p = 0.008**	58.91 (36.12–102.54)	45.63 (29.03–77.19)	42.41 (28.22–82.82)	46.15 (27.54–63.72)	**p = 0.006**	**p = 0.025**
MAM-36	94.00 (73.00–115.00)	107.00 (77.00–129.00)	111.00 (102.00–130.00)	110.50 (86.25–124.25)	**p = 0.000**	p = 0.875	106.00 (91.00–124.50)	108.00 (94.00–129.50)	117.00 (89.00–128.50)	123.00 (104.00–135.00)	p = 0.463	**p = 0.008**
COPM												
Performance	24.00 (15.00–28.00)	NA	29.00 (25.00–33.00)	30.00 (22.50–34.50)	**p = 0.006**	p = 0.128	19.00 (13.50–24.00)	NA	20.00 (16.50–30.00)	28.00 (17.00–34.00)	p = 0.052	p = 0.044
Satisfaction	24.00 (18.00–31.00)	NA	30.00 (22.00–35.00)	31.00 (21.00–40.00)	**p = 0.005**	p = 0.041	19.00 (11.00–26.50)	NA	20.00 (16.00–26.00)	27.50 (13.75–39.75)	**p = 0.018**	p = 0.081
SF-36												
Health change	25.00 (0.00–50.00)	25.00 (0.00–50.00)	25.00 (0.00–50.00)	25.00 (0.00–56.25)	p = 0.272	p = 1.000	25.00 (0.00–50.00)	25.00 (12.50–50.00)	50.00 (0.00–50.00)	25.00 (0.00–50.00)	p = 0.366	p = 0.317
Physical functioning	50.00 (25.00–80.00)	50.00 (45.00–90.00)	50.00 (40.00–90.00)	47.50 (35.00–80.00)	p = 0.131	p = 0.831	55.00 (32.50–70.00)	50.00 (32.50–80.00)	65.00 (37.50–80.00)	70.00 (50.00–85.00)	p = 0.285	p = 0.317
Role functioning/physical	25.00 (0.00–75.00)	50.00 (0.00–75.00)	25.00 (0.00–75.00)	37.50 (0.00–81.25)	p = 0.719	p = 0.633	25.00 (0.00–75.00)	25.00 (25.00–50.00)	25.00 (0.00–50.00)	50.00 (0.00–75.00)	p = 0.314	p = 0.102
Role functioning/emotional	100.00 (67.00–100.00)	100.00 (66.70–100.00)	100.00 (33.33–100.00)	100.00 (58.35–100.00)	p = 0.681	p = 0.510	100.00 (33.00–100.00)	100.00 (33.30–100.00)	66.67 (33.33–100.00)	100.00 (33.30–100.00)	p = 0.513	p = 1.000
Energy/fatigue	70.00 (65.00–85.00)	70.00 (55.00–85.00)	65.00 (50.00–85.00)	75.00 (53.75–86.25)	p = 0.193	p = 0.146	60.00 (47.50–75.00)	65.00 (47.50–77.50)	65.00 (47.50–82.50)	75.00 (50.00–100.00)	p = 0.752	p = 0.683
Emotional well-being	84.00 (72.00–92.00)	88.00 (64.00–96.00)	76.00 (52.00–92.00)	80.00 (67.00–94.00)	p = 0.068	p = 0.073	72.00 (60.00–84.00)	80.00 (58.00–84.00)	80.00 (60.00–88.00)	84.00 (60.00–100.00)	p = 0.136	p = 0.465
Social functioning	75.00 (63.00–100.00)	100.00 (62.50–100.00)	87.50 (62.50–100.00)	100.00 (59.38–100.00)	p = 0.360	p = 0.518	63.00 (38.00–88.00)	62.50 (62.50–87.50)	62.50 (50.00–87.50)	75.00 (50.00–87.50)	p = 0.868	p = 0.581
Pain	68.00 (45.00–80.00)	77.50 (55.00–100.00)	77.50 (55.00–90.00)	77.50 (56.88–100.00)	p = 0.420	p = 0.529	68.00 (47.50–95.00)	77.50 (51.25–100.00)	77.50 (51.25–100.00)	77.50 (67.50–100.00)	p = 0.582	p = 0.414
General health	60.00 (45.00–80.00)	70.00 (35.00–90.00)	65.00 (45.00–85.00)	50.00 (45.00–78.75)	p = 0.255	p = 0.222	60.00 (37.50–75.00)	60.00 (50.00–77.50)	60.00 (40.00–77.50)	65.00 (40.00–85.00)	p = 0.509	p = 1.000
MFIS	26.00 (8.00–36.00)	21.00 (2.00–40.00)	25.00 (5.00–39.00)	22.50 (3.75–32.50)	p = 0.879	p = 0.944	27.00 (20.50–35.50)	33.00 (21.50–36.50)	33.00 (18.00–39.00)	16.00 (13.00–38.00)	p = 0.410	p = 0.352
TIS	15.00 (13.00–19.00)	18.00 (16.00–22.00)	20.00 (17.00–22.00)	22.00 (16.00–23.00)	**p = 0.000**	p = 0.527	14.00 (12.50–18.50)	17.00 (13.50–21.00)	17.00 (13.00–22.00)	21.00 (17.00–23.00)	**p = 0.014**	p = 0.102
AROM												
Abduction	81.00 (66.00–124.00)	88.00 (77.00–138.00)	95.00 (83.00–119.00)	110.00 (84.75–143.50)	**p = 0.002**	p = 0.064	118.00 (73.50–146.00)	119.00 (68.00–149.50)	114.00 (70.00–146.00)	134.00 (116.00–160.00)	p = 0.396	p = 0.154
Flexion	94.00 (78.00–132.00)	104.00 (90.00–131.00)	107.00 (94.00–132.00)	120.50 (101.50–141.25)	**p = 0.006**	p = 0.076	120.00 (87.00–146.50)	122.00 (107.50–147.50)	123.00 (101.00–153.50)	130.00 (122.00–162.00)	p = 0.236	**p = 0.013**

Data represented as median (interquartile range). p-values from Wilcoxon signed-rank test (2-sided-, significant *p*-values are indicated in bold font. *WMFT* Wolf Motor Function Test, *FAS* Functional Ability Scale, *MAM-36* Manual Ability Scale, *COPM* Canadian Occupational Performance Measure, *SF-36* Short Form-36, *MFIS* Modified Fatigue Impact Scale, *TIS* Trunk Impairment Scale, *AROM* Active Range of Motion

The stroke group (n = 36) consisted of 24 male and 12 female persons with stroke, with a mean age of 62.06 ± 15.05. Seventeen participants were allocated to the control group (10 male and 7 female participants, mean age 62.59 ± 15.58), while 19 persons were allocated in the intervention group (14 male and 5 female participants, mean age 61.58 ± 14.97). In the stroke group, no significant differences were found in the baseline characteristics and primary outcome measures between control group and intervention group.

Regarding *within group differences*, similar results were found in the stroke group compared to the general group, with the exception of AROM-flexion, as no significant difference was found in the control group.

When examining the specific period of training (T0-T2) and follow-up (T2-T3), similar results were found in the stroke group as opposed to the general group during training period. The results within the stroke intervention group are similar to the results in the intervention group of the general group; All outcome measures that showed significant improvement in the general intervention group, also show significant improvement in the stroke intervention group. As for the stroke control group, the same outcome measures showed significant improvement except for the COPM-performance, which did not show significant improvement (p = 0.052) compared to the general control group. Looking into the follow-up period, similar results are found for the stroke intervention group compared to the general intervention group, with the exception of AROM-abduction where no significant improvement is found (p = 0.064). The control group of the stroke group is not very similar to the general group. For the stroke control group, significant improvement is found for the MAM-36 (p = 0.008) and AROM-flexion (p = 0.013), but significant deterioration was found for WMFT-Time (p = 0.025) as the performance time was higher at follow-up. Also similar to the general group is that no significant differences in training period as well as follow-up period were found in SF-36 and MFIS in both intervention or control group.

Regarding the *between group difference*, similar results were found for the stroke group as also a significant improvement was found in MAM-36 in favour of the intervention group during training period (p = 0.000, r = 0.591) and in favour of the control group during the follow-up period (p = 0.019, r = − 0.373). Regarding the secondary outcome measures, significant improvement was found in favour of the intervention group in TIS (p = 0.011, r = 0.421) during training period.

## Discussion

The aim of this study was to investigate whether the use of i-ACT as an additional tool has an effect on functional ability and performance, quality of life (QoL), fatigue, trunk movement, and shoulder active range of motion (AROM).

No major differences were found between the intervention group and control group on any of the outcome measures for both the general group as well as the stroke group. Both the intervention and control groups improved over time on the primary outcome measures (i.e. WMFT, MAM-36, and COPM). This is in concordance with other studies that used virtual reality, exergames, or robot-assisted therapy, who found similar results – i.e. they did not find significant differences on functional ability between intervention group and control group [8, 46–48]. But studies showed an increase in motivation when using virtual reality of games [1, 48, 49] and also suggest that the use of virtual reality or virtual games may be beneficial in improving ADL when provided as additional training [46]. As seen in the results, we found significant differences between baseline and cessation of training, but not as much as between cessation of training and follow-up. Whether this suggests that additional therapy with i-ACT has, although small, a positive influence on the functional and occupational performance of persons with deficits, needs to be further investigated.

We expected a significant difference regarding the COPM results, but no significant differences between intervention and control group were found. On the one hand the COPM results in the intervention groups were already high at baseline, but there was still an increase during the training period. On the other hand the variance was large in both intervention and control groups. We did find a clear distinction between the intervention group and control group regarding the amount of trained personal goals. Although conventional therapy also focusses on providing a client-centred approach, our results suggest that i-ACT can support a client-centred approach as about 88% of the persons’ goals were implemented in i-ACT training compared to about 46% in control group during conventional therapy. This finding confirmed our third hypothesis as i-ACT is specifically developed to incorporate a client-centred approach by being able to record and set different kinds of activities (which are determined by the person with deficits), and provide individualised real-time feedback regarding the performance of the person with deficits [13]. These features distinguish i-ACT from other Kinect-based systems which mostly use commercially available (exer)games, which are not designed to meet rehabilitation goals such as feedback on compensation strategies, coordination patters, etc. [1, 2, 4, 5, 21, 22]. Using the COPM or other goal setting tools that focus on person’s goals and involvement are important aspects of client-centred therapy and increases therapy motivation and also adherence [7, 9–12]. In this study, we found a very high adherence towards i-ACT training despite voluntary participation, i.e. 97.92%, which confirms the results from our cohort study [24]. This percentage might have been even higher because the reasons to miss an i-ACT therapy session were doctor appointments, hospital visits and family-related activities (i.e. family birthday party). Also, participants commented that they liked exercising with the i-ACT and that it gave them a feeling of involvement by explicitly mentioning the goals they provided during the intake with COPM.

Regarding the secondary outcome measures (i.e. QoL measured by SF-36, fatigue measure by MFIS, trunk function measured by TIS, and shoulder AROM), no significant/evident trends were found. For QoL, the reason might be that the intervention was too short to be of influence on the QoL of persons with CNS decifits or the influence was too small to be detected by the used measurement (i.e. SF-36). With regard to shoulder AROM and TIS, these were not the main focus of the intervention but we hypothesised a possible secondary improvement. Although no significant improvement regarding fatigue was found, no increase in fatigue was found either. This outcome suggests that the additional training with i-ACT is very tolerable by persons with CNS deficits.

In this study, the intervention consisted of 3 × 45 min/week of exercises with i-ACT for 6 weeks. The aim was to provide 45 min of extra training. This goal was achieved, but more training time was not possible due to the available time in the different participating centres. Although other studies with similar or slightly higher dosage show the same results, i.e. improvement within groups but no significant difference between intervention and control group [3, 50–52], dosage seems an important factor within motor learning and neuroplasticity [53–56]. Lang et al. (2016) describe the dosage of training as a combination of four aspects, i.e. frequency (sessions per week), duration (time period of intervention), amount of practice (by number of repetitions or minutes of active therapy), and level of difficulty [53]. Although Lang et al. (2016) found no significant dose–response effect [53], other evidence still suggests that higher dosage is better [57–61]. Taking this into account, the dosage in our study was probably too low to find significant differences and we recommend performing an intervention with a higher dosage of therapy. The most common dosage would be at least one hour/session, 5d/week, 4–6 weeks as based on the meta-analysis by Saposnik et al. (2011), which found 11 of 12 studies showing significant improvement toward virtual reality therapy for selected outcomes such as WMFT [4]. We did not find increased fatigue, as measured by MFIS, based on the current intensity. Therefore, there are no contraindications to increase dosage. Future research should be performed with increased intensity to investigate the benefits of increased training with i-ACT on functional ability and performance, but also towards neuroplastic changes.

By taking into account the four aspects of dosage by Lang et al. [53], the i-ACT can provide an added value to register the dosage of training with i-ACT as it registers number of sessions (i.e. frequency), total training time (i.e. duration and frequency), exercise time (i.e. amount of practice), amount of repetitions (amount of practice), number of targets reached (from which a therapist can derive the level of difficulty), and the percentage of compensational movements (from which a therapist can derive the level of difficulty). i-ACT also has the potential to increase therapy dosage without major financial burden and negative side events as no adverse events or increase of fatigue were reported towards the additional use of i-ACT, no interference of additional i-ACT training to conventional care is found, and i-ACT is considered a low-cost system [24]. Also, during the COVID-19 pandemic, a secured online platform is developed so all results can be consulted by therapists from a distance which creates opportunities for the independent use of i-ACT in rehabilitation or even implementation in the person’s home environment.

This study may have suffered from insufficient power which might have contributed to the lack of significance between groups. Furthermore, missing data occurred in the follow-up data and therefore conclusions based on the follow-up period (T2-T3) have even less power and must be treated with caution.

Other studies with rehabilitation technology in neurorehabilitation, have similar numbers of participants [1, 8, 48, 52, 62–65]. Furthermore, the sample consisted of a very diverse population to generalise towards a broader spectrum within neurorehabilitation, but mainly persons with stroke. A comparison of stroke versus general group, and stroke versus other CNS deficits was performed but similar results were found between the general group and stroke group as well as the stroke group versus other CNS deficits. Considering the similar results, there is no reason to expect that i-ACT does not work within all these different groups. But certainty is only guaranteed when research is performed in separate target groups. Future research could also assess implications of additional i-ACT training on participation level, preferably linked with the provided COPM goals, with a long term monitoring.

## Conclusions

Upper limb functional ability and perceived performance on ADL improved after 6 weeks of training, in both the i-ACT intervention group and control group. Although the use of i-ACT did not seem to have a significant added value with regards to functional outcome over conventional therapy, i-ACT may provide opportunities for therapists to supply persons with deficits with additional client-centred task-oriented therapy with individualised performance feedback. For this purpose, the dosage of therapy with i-ACT needs to be increased in future research and therapy.

## Data Availability

The datasets generated and analysed during are available from the corresponding author on reasonable request.
